# Properties and Degradation of Novel Fully Biodegradable PLA/PHB Blends Filled with Keratin

**DOI:** 10.3390/ijms21249678

**Published:** 2020-12-18

**Authors:** Katarína Mosnáčková, Alena Opálková Šišková, Angela Kleinová, Martin Danko, Jaroslav Mosnáček

**Affiliations:** 1 Polymer Institute, Slovak Academy of Sciences, Dubravská Cesta 9, 845 41 Bratislava, Slovakia; alena.siskova@savba.sk (A.O.Š.); angela.kleinova@savba.sk (A.K.); martin.danko@savba.sk (M.D.); 2 Centre for Advanced Materials Application, Slovak Academy of Sciences, Dubravská Cesta 9, 845 11 Bratislava, Slovakia

**Keywords:** keratin, natural waste material, biodegradable composites

## Abstract

The utilization of keratin waste in new materials formulations can prevent its environmental disposal problem. Here, novel composites based on biodegradable blends consisting of poly(lactic acid) (PLA) and poly(3-hydroxybutyrate) (PHB), and filled with hydrolyzed keratin with loading from 1 to 20 wt % were prepared and their properties were investigated. Mechanical and viscoelastic properties were characterized by tensile test, dynamic mechanical thermal analysis (DMTA) and rheology measurements. The addition of acetyltributyl citrate (ATBC) significantly affected the mechanical properties of the materials. It was found that the filled PLA/PHB/ATBC composite at the highest keratin loading exhibited similar shear moduli compared to the un-plasticized blend as a result of the much stronger interactions between the keratin and polymer matrix compared to composites with lower keratin content. The differences in dynamic moduli for PLA/PHB/ATBC blend filled with keratin depended extensively on the keratin content while loss the factor values progressively decreased with keratin loading. Softening interactions between the keratin and polymer matrix resulted in lower glass transitions temperature and reduced polymer chain mobility. The addition of keratin did not affect the extent of degradation of the PLA/PHB blend during melt blending. Fast hydrolysis at 60 °C was observed for composites with all keratin loadings. The developed keratin-based composites possess properties comparable to commonly used thermoplastics applicable for example as packaging materials.

## 1. Introduction

The interest for fully biodegradable “green” materials in society has increased, mainly due to the accumulation of plastic waste in landfills and its penetration and contamination in the form of microplastics into the whole ecosystem. Biodegradable polymers represent materials which easily undergo enzymatic hydrolysis, and they are prone to natural recycling by biological processes. Among these polymeric materials, the aliphatic polyesters are the most attractive because of their good mechanical properties, processability and the ability to undergo both the hydrolytic degradation and biodegradation by soil microorganism in compost [1]. Poly(hydroxyalkanoates) (PHAs) and poly(lactic acid) (PLA) represent biodegradable polymers derived from renewable resources, commercialized at a large scale. Great attention has been paid to PLA due to its thermoplastic behavior, biocompatibility and physical properties, and the ability to tune these properties by the addition of plasticizers (glycerol, triacetine, PEG, citrate esters, oils) [2,3,4,5,6], fillers (clay [7,8], carbon black [9,10], silica [11,12,13]), compatibilizers (tri-block copolymer PLA-PBAT-PLA [14], PLA-g-PEG [15]) or polymers (PHB [1,16,17,18], PBAT [19]). These additives lead to its increasing applicability in many industries, such as packaging [18,20,21], agriculture [1,16,22] and medicine [23,24,25]. In addition, the incorporation of functional fillers in the PLA matrix could significantly improve the physical properties as well as the structural characteristics that play key roles in various applications, for example in tissue engineering [26]. In addition, the blending of PLA with another polymer at certain weight composition can lead to the synergistic effect resulting in greatly improved mechanical properties, similarly to Armentano et al. [27].

Keratin is a natural biomaterial derived from wool and represents 95% of the dry matter [28]. Wool is quite resistant to the attack of microorganisms, which are able to breakdown the keratinous fiber only in hydrophilic conditions. Crude sheep wool is classified as long-term degradation waste. While it is organic and will break down over time, the process at the central composting facility is not designed for wool. Therefore, its high production, connected with sheep breeding, represents an environmental problem and unprocessed wool cannot be left in nature or compost, like waste from renewable resources, but must be disposed of by a special way, which consists of quite expensive procedures [29]. One option is the isolation of wool keratin to a form similar to the native one without any loss of its functionality. Besides carboxyl and thiol groups, the keratin hydrolyzed from wool contains amino acids responsible for its biodegradability, biocompatibility and bioactivity. However, the functionality as well as microstructure depends ultimately on the keratin extraction conditions such as type of used medium (reduction, oxidation or sulfitolysis) or reaction time [30]. A crucial parameter is its hard processing due to keratins α-helical structure with strong intermolecular interactions reflecting in low solubility in common solvents. This causes a worse ability to disperse keratins homogeneously into the polymer matrix, thus considerably affecting its applicability [31]. Despite that, keratin found a place in many applications as a separator for membrane devices [32,33,34] in biomedicine as tissue scaffolds [35,36], as flame retardants [37,38] and photoprotective coating [39].

So far, only a few studies used the keratin as a potential functional filler for biodegradable polymers, despite the fact that it bears rich functionality in the form of amino, thiol and carbonyl groups. Lagaron et al. [40] mixed poly(3-hydroxybutyrate-co-3-hydroxyvalerate) (PHBV) with feather keratin by melt compounding, varying the keratin loading from 1 to 50 wt %. It was shown that addition of 1 wt % exhibited a good interphase interaction reflecting in an enhanced mechanical performance and about 50% reduction in oxygen permeability compared to neat matrix. However, authors also reported that higher contents of keratin—more than 10 wt % were detrimental to most of the properties (transparency, water permeability, elastic modulus). Similarly, Wang et al. [41] electrospun PHBV blends with keratin and the prepared nanofibrous mats were tested for cell compatibility. The results showed an enhanced cytocompatibility of keratin electrospun mats in comparison with neat PHBV. The wool keratin was also used in study of Li et al. [42] for preparation of electrospun hydroxyapatite-poly(L-lactic acid) fibrous membrane. The formed membrane induced significant bone formation compared to neat electrospun PLA as a result of the strong interactions between the keratin and Ca^2+^ cations of HA.

Su et al. [43] produced novel composites based on poly(ε-caprolactone)/polyethyleneoxide loaded with hyaluronic acid and keratin by emulsion and coaxial electrospinning methods. Cytotoxicity test of this spun mats shows increased in cell viability and cell proliferation and demonstrated its efficient for wound healing applications. Huda et al. [44] similarly to Darie et al. [45] used for improving the mechanical properties of PLA poultry feather keratin. The authors showed that the addition of feather keratin slightly improved the mechanical properties (tensile moduli), especially at lower keratin loading. Arranberi et al. [46] reported preparation of multicomponent system based on PLA/polybutyrate adipate terephthalate (PBAT) with much higher contents of chicken feather fibers (up to 60 wt %). It was shown that, higher loading of chicken feather fibers negatively affected the thermal stability and mechanical properties due to extensive heterogeneity of material.

In this study, we report the preparation and characterization of plasticized PLA/PHB blend (85:15) filled with hydrolyzed keratin originated from sheep wool. The amount of keratin added to the polymer matrix varying from 1 to 20 wt %. Prepared keratin materials were tested in the terms of mechanical, thermal and rheological properties as well as their hydrolytic degradation. Wool keratin extracted through acidic hydrolysis was found to be a suitable filler for melt blending with PLA/PHB when acetyl tributyl citrate (ATBC) was used as plasticizer.

## 2. Results and Discussion

### 2.1. Keratin Composites Preparation and Characterization

Following actual trends, in fact, there is not always an emphasis on preparation of the materials with superior mechanical properties but rather on environmentally friendly and/or recycling materials retaining at least some mechanical properties sufficient for targeted, many times disposable, applications in packaging, agriculture or medicine. Another important aspect is the use of material from biowastes which do not decompose freely in nature. In the case of keratin there is a big problem with its disposing due to financial aspects, but also with its repeated use without processing. In order to achieve easier processability, here the keratin was first hydrolyzed under acidic conditions as described elsewhere [47,48].

As already mentioned, various authors described good properties of keratin-based composites at very low loading, but deterioration of the mechanical and thermal properties at keratin loadings of about 10 wt %. Therefore, in order to investigate effect of keratin on properties of PLA/PHB 85:15 blend, we prepared PLA/PHB/keratin composites with keratin loading of 1 and 10 wt %. In addition, in order to improve compatibility of the keratin with the blend the ATBC was used as plasticizer. It was already shown in our previous works, that using of ATBC in PLA/PHB blends in ratio of PLA/PHB/ATBC 85:15:15 significantly improved the chains mobility due to higher free volume in the material and thus increased the elongation at break of the PLA/PHB blend in few orders of magnitude [1,16,17]. Totally 5 keratin PLA/PHB/ATBC composites were prepared with the keratin loadings of 1, 3, 5, 10 and 20 wt %.

**Figure 1:**
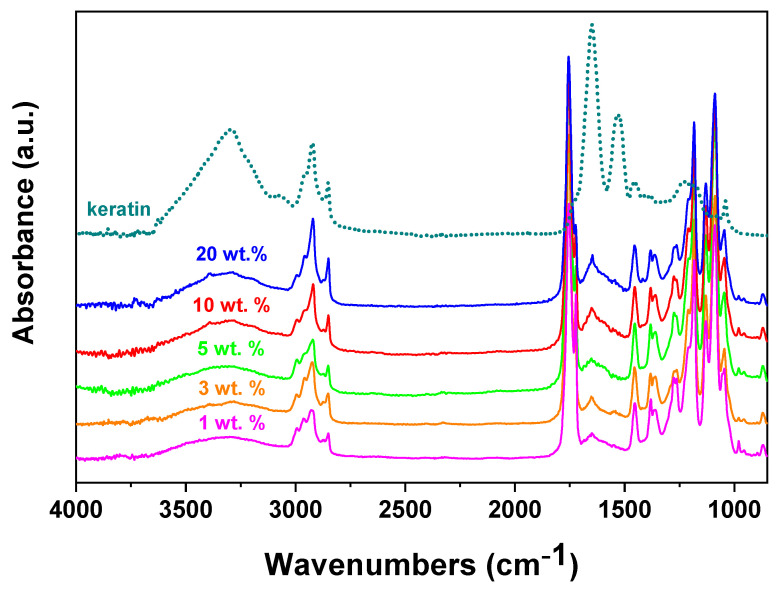
FTIR-ATR spectra analysis of keratin (dotted line) and PLA/PHB/ATBC composites with keratin loading from 1 to 20 wt %. PLA, poly(lactic acid); PHB, poly(3-hydroxybutyrate; ATBC, acetyl tributyl citrate.

The increase of keratin in the composites could be followed by ATR spectra of PLA/PHB/ATBC filled with various keratin loading (Figure 1). A broad band located between 3650–3050 cm^−1^ can be assigned to the valency vibrations of the OH– and NH functional groups of the keratin. In addition, in the area of –CH groups the keratin shows the maximum at 2920 cm^−1^. The characteristic strong peaks attributed to deformation vibrations of the amide groups can be seen at 1650 and 1550 cm^−1^ from C=O stretches and N–H bending, respectively [47,49]. These typical peaks of the keratin were detected in all filled samples spectra and the intensity of the peaks increased with keratin loading as a proof of keratin presence and its incorporation to the polymer matrix.

### 2.2. Properties of the Keratin Composites

**Figure 2:**
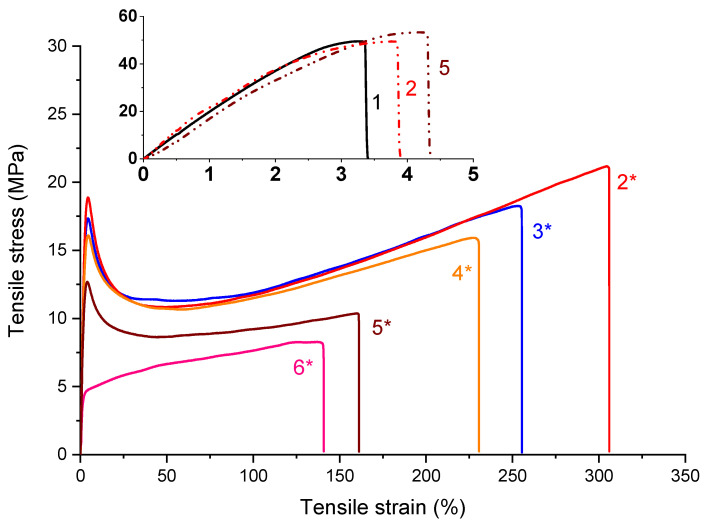
Stress–strain curves for PLA/PHB/ATBC filled with (2*) 1 wt %, (3*) 3 wt %, (4*) 5 wt %, (5*) 10 wt % and (6*) 20 wt % of the keratin. The inset shows the stress–strain curve of PLA/PHB blend (1) unfilled and filled with (2) 1 wt % and (5) 10 wt % of keratin.

The mechanical properties of the polymers and composites are usually strongly dependent on both the filler loading and addition of plasticizers. Here the mechanical properties were first investigated by tensile test measurements. The stress–strain curves for PLA/PHB blend and all the prepared composites are shown in Figure 2. As can be seen from the inset of Figure 2, the unfilled PLA/PHB shows brittle behavior similarly observed for both composites filled with 1 and 10 wt % of the keratin. The presence of filler slight affected the strain depending on keratin content while the tensile strength remained unchanged.

Addition of either 1 or 10 wt % of the keratin (shown in Figure 2) led to slightly progressive increase in elongation at break indicating higher mobility of the polymer chains. In contrast to that, addition of the plasticizer to the keratin composites led to significant increase in mobility of the polymer chains accompanied by one to two orders increased elongation at break. The significant differences in behavior upon stress were observed mainly at the highest keratin loading, resulting in the disappearance of the initial maxima of tensile strength represented as yield strength due to the more elastic structure of materials. Besides the sample with the highest keratin loading, a decrease in both elongation at break and tensile strength with an increased keratin loading was observed without significant changes in the stress–strain slope. Many authors reported [50] higher keratin loading (larger than 10 wt %) leading to significant deterioration of tensile properties, due to worse compatibility and disintegration into the material.

**Figure 3:**
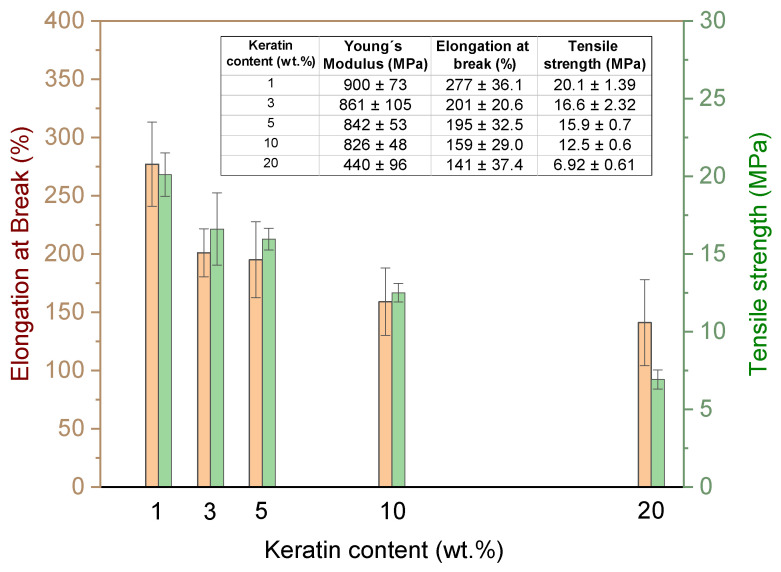
Changes in tensile properties (*E*, *ε*_B_, *σ*_M_) for (PLA)/PHB/ATBC blend before and after addition of different amount of keratin varying from 1 to 20 wt % loading.

Elongation at break (*ε*_B_) and tensile strength (*σ*_M_) of PLA/PHB/ATBC/keratin composites are shown in Figure 3 and the values are summarized together with the values of Young’s modulus (*E*) in the table (inset of Figure 3). With increasing keratin loading, progressive degrease in both *ε*_B_ and *σ*_M_ was observed reflecting in better malleable and worse plasticity as a consequence of limited chain mobility in the amorphous region leading to insufficient options for chain straightening under tension.

It is important to note that the PLA/PHB/ATBC blend filled with the 20 wt % of the keratin still showed extraordinary mechanical properties of approximately 140% elongation at break and four times lower tensile strength compared to original PLA/PHB blend as a result of more flexible structure.

**Figure 4:**
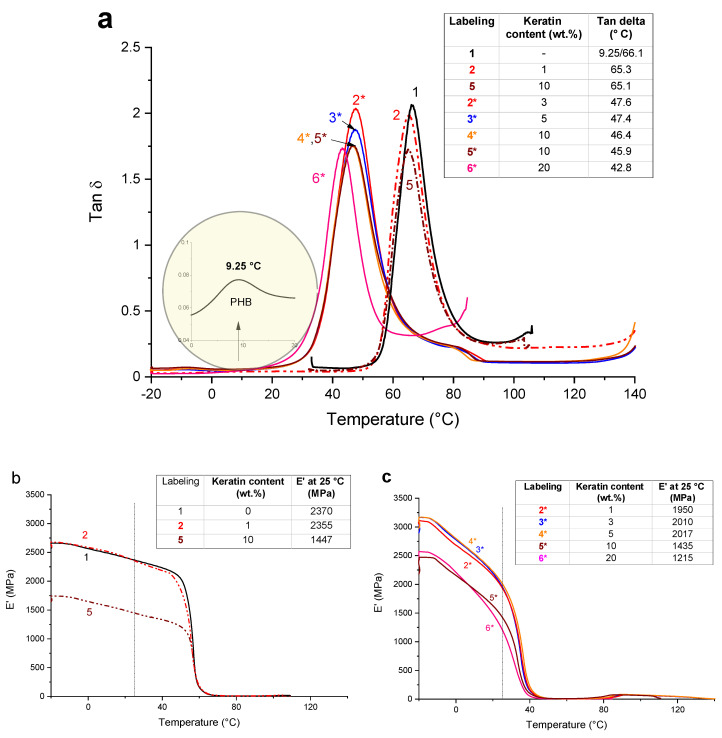
Evolution of (**a**) loss factor (*tan δ*) and (**b**), (**c**) storage moduli (*E*′) with temperature for PLA/PHB before and after addition of 1 wt % and 10 wt % keratin and plasticized PLA/PHB/ATBC blend filled with 1 wt %, 3 wt %, 5 wt %, 10 wt % and 20 wt % of the keratin.

The dynamic mechanical thermal analysis is a sensitive characterization technique for obtaining additional information at the level of multiphase polymer systems. The total response strongly depends on the phase or molecular structure within the polymer such as crosslinking, crystallinity and branching. The behavior of storage modulus (*E*′) and loss factor (tan δ) for the PLA/PHB blend and all prepared composites before and after addition of plasticizer (ATBC) are compared in Figure 4. The non-plasticized PLA/PHB blend exhibits two peaks assigned to glass transition temperature (*T_g_*) of PHB and PLA because of immiscible polymer mixture [16]. The presence of the keratin affected *T_g_*, which was surprisingly slightly shifted to the lower temperatures, and also reduced the height of loss factor peak as a consequence of higher free volume leading to enhanced chain mobility. The addition of ATBC reflected in a typical plasticizing effect resulting in significant shift of *T_g_* to lower values, while the trend of decrease of loss factor maximum with increasing the keratin content was retained. Obviously, the presence of protein-like structure can extensively influence the mobility of the polymer chain due to acting as a barrier for relaxation processes needed for arrangement of the polymer chains. In accordance a progressive decrease in *T_g_* with increased keratin content was observed as a consequence of higher free volume which is usually observed as results of plasticizing.

Figure 4b,c shows the dependence of storage modulus (*E′*) on temperature for all the prepared samples. The observed changes in *E′* are usually related to the interface adhesion between polymer phases and filler particles, as well as can be assigned to material degradation, crystallinity or crosslinking. Addition of 1 wt % of keratin into the PLA/PHB blend led to increased value of *E′* at 25 °C reflecting pronounced stiffness as a result of reinforcing effect. Increasing keratin content to 10 wt % led to dramatic decrease of *E′* at 25 °C indicating higher mobility of the chains leading to softer material. The addition of ATBC led to a sharp decline in *E′* at room temperature as a result of plastification of the material and reduction of the strong inter-chain interactions that promote enhanced chain mobility reflecting in higher material flexibility. The character of *E′* is strongly dependent on the keratin loading and showing dual behavior similarly as observed at composites without ATBC. The keratin loading above 10 wt % led to significant *E′* decrease both at 25 °C as well as in glassy state, compared to the composites with low keratin loadings. In glassy state, however, the *E′* values of plasticized composites are higher than *E′* values for the unplasticized ones, indicating softening of the material.

It is well known that rheological properties are very important from the point of view of polymer processing and its feature applications. In order to investigate the influence of keratin on the rheological properties of composites in melt conditions, the oscillatory shear measurements were performed. The viscoelastic properties of composites usually depend on particle size, shape, concentration and particle distribution in polymer matrix [51], therefore the rheology was used as a tool for evaluation of the phase interactions and structural evolution of polymer/keratin composite in polymer melt. First the strain sweep tests were done to find the presence of stable viscoelastic region for neat PLA/PHB blend as well as for all keratin composites. Subsequent frequency sweeps were carried out within the linear viscoelastic region.

**Figure 5:**
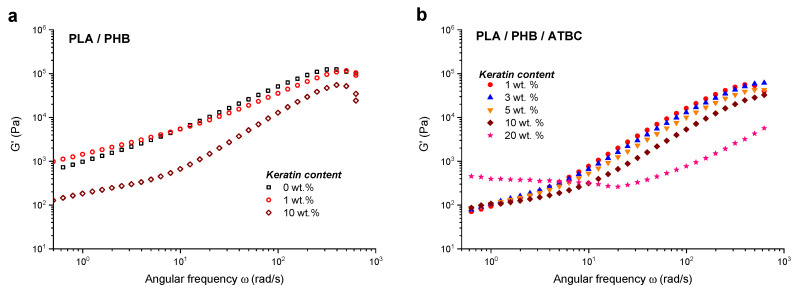
Dependencies of storage modulus *G*′ vs. frequency for (**a**) neat PLA/PHB blend without keratin and with 1 wt % and 10 wt % of keratin loading and (**b**) PLA/PHB/ATBC blend with 1 wt %, 3 wt %, 5 wt %, 10 wt % and 20 wt % of keratin loading.

As seen from Figure 5, except for the composite with 20 wt % of the keratin, an increase in the *G*′ value with the increasing frequency was observed for all studied samples. The explanation is that at low frequency the duration is large and the chains can be easily re-oriented while at higher frequencies the entangled chains or coils had less time for relaxation, thus leading to an increase of the moduli.

The addition of ATBC significantly affected the frequency dependence of the storage/loss modulus in the full frequency scale and a massive decrease of approximately one order of magnitude was observed as a result of an effective plasticizing of the stiff and relatively brittle unplasticized PLA/PHB blend and composites. At lower frequencies, a slight progressive increase of *G*′ was observed with an increase of keratin content up to 10 wt % in the plasticized samples (Figure 5b). A further increase of keratin loading to 20 wt % led to an increase of *G*′ values in approximately one order of magnitude with a broad plateau at the frequencies up to approximately 20 rad/s. With the keratin content increase also the number of interactions within the polymer matrix increase and the spatial filler network structures are formed. Chodak et al. [52] reported that the interactions between heterogeneous structures such as organomodified montmorillonites resulted in an appearance of apparent yield stress characterized by the plateau of *G*′ at low frequencies. This is in good agreement with the results observed here for keratin composites, especially at the highest keratin loading of 20 wt %.

**Figure 6:**
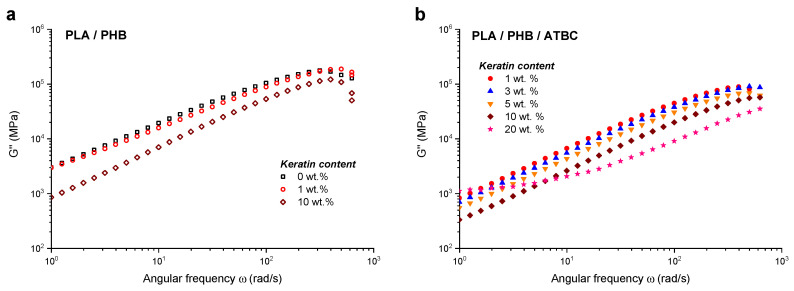
Dependencies of loss modulus *G*″ vs. frequency for (**a**) neat PLA/PHB blend without keratin and with 1 wt % and 10 wt % of keratin loading and (**b**) PLA/PHB/ATBC blend with 1 wt %, 3 wt %, 5 wt %, 10 wt % and 20 wt % of keratin loading.

The loss moduli of the composites decreased with increasing level of incorporated keratin (Figure 6.) due to reduction of energy loss dissipated directly into heat. Except for the composite with 20 wt % of the keratin, the character of *G*″ slopes was essentially similar for all the samples. Darie et al. [45] deduced that polymer chains dynamics at the scales comparable to the entanglement length were not affected by presence of filler particles, based on the similar results of the *G*″ decrease observed in the case of PLA composites filled with chitosan/keratin. Zhang et al. [53] studied the effect of glycerol monostearate on shear moduli and complex viscosity of PLA and showed that increasing concentration of low molecular plasticizer significantly improved ductility of PLA resulting in decreased *G*′, *G*″ moduli and complex viscosity (η*).

**Figure 7:**
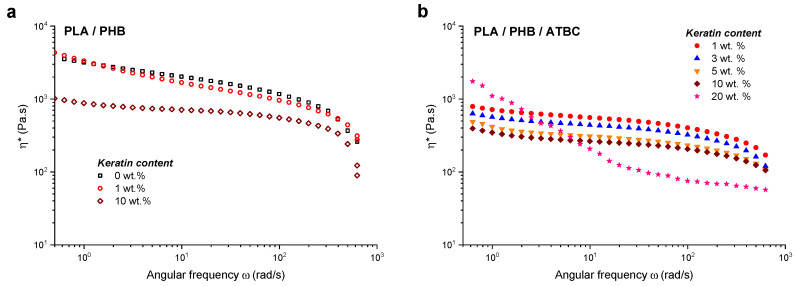
The dependencies of (η*) on angular frequency *ω* for (**a**) neat PLA/PHB blend without keratin and with 1 wt % and 10 wt % of keratin loading and (**b**) PLA/PHB/ATBC blend with 1 wt %, 3 wt %, 5 wt %, 10 wt % and 20 wt % of keratin loading.

Figure 7 shows the frequency dependences of η* for neat PLA/PHB blend and all keratin composites, unplasticized and plasticized. The η* values of un-plasticized composites were of about half order of magnitude higher compared to plasticized ones. A complex viscosity plateau region in the frequencies range of about 5–50 rad/s along with a decreasing viscosity trend at higher frequencies was observed for all plasticized samples besides the composite with the 20 wt % of keratin loading. With increasing keratin loading the η* frequency values slightly decreased s. Completely different η* behavior was observed for the composite at the highest keratin concentration, characterized by sharp decline in the low frequency region followed by plateau at higher frequencies indicating a problem with relaxation already at the lower frequencies, most probably due to the existing physical network within the composite.

### 2.3. Visualisation by Scanning Electron Microscopy

**Figure 8:**
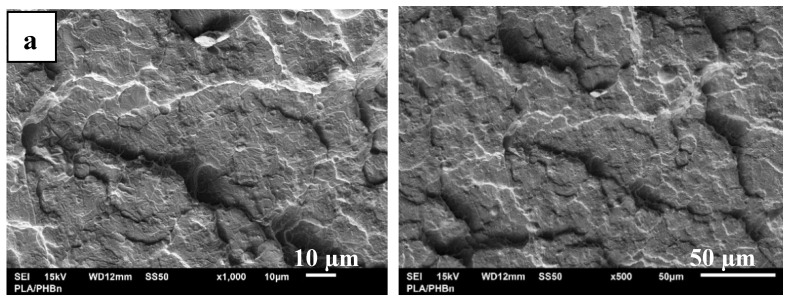

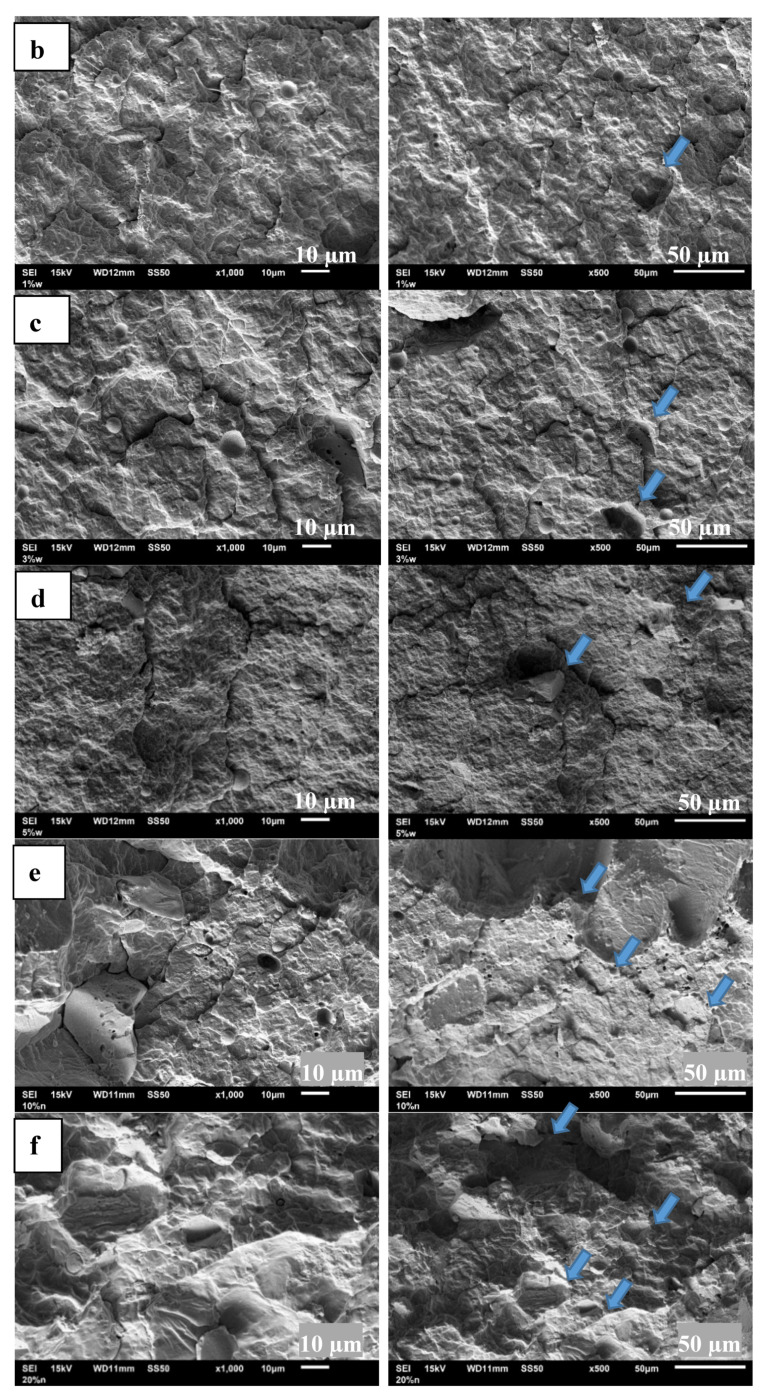
Scanning electron micrograph of: (**a**) PLA/PHB blend, (**b–f**) PLA/PHB/ATBC composites with different keratin content. Micrographs are labelled according to keratin content, i.e., **b**, **c**, **d**, **e**, **f** contain 1, 3, 5, 10 and 20 wt % of keratin, respectively. SEM images were taken at 500× (**right side**) and at 1000× (**left side**) magnification. Arrows in the left micrographs show some of the visible aggregates of the keratin.

The SEM images of PLA/PHB surfaces after brittle fracture are shown in Figure 8. After addition of keratin at 1 wt % loading the presence of globular particles with diameter approximately 5 µm are clearly visible at 500× as well as 1000× magnification. At higher keratin loading the evidence of the wide range of particle sizes formed in the material due to agglomeration were observed. For the composites, up to 5 wt % of filler loading the incorporated keratin forms the globuli with small cracks distributed over the whole surface, at higher loadings (10 and 20 wt %) the filler is arranged as rock shaped structures with diameters varying from 5 to 100 µm. The absence of cracks in the PLA/PHB/ATBC composites with higher keratin contents (≥10 wt %) can be attributed to the significantly lower values of tensile strength compared to the composites filled with lower keratin loading, indicating the more compact and flexible structure. The results are in good agreement also with the dynamic mechanical thermal analysis (DMTA) results showing the significant storage moduli decline resulting in better elasticity of the system at higher keratin loading.

### 2.4. Degradation of Keratin Composites

**Figure 9:**
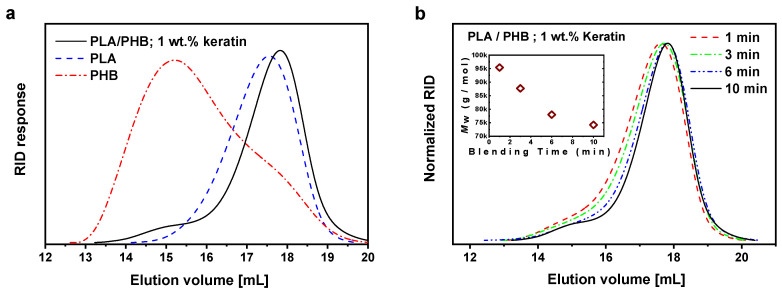
(**a**) GPC traces of original PLA, original PHB and prepared PLA/PHB composite filled with 1 wt % of keratin and (**b**) evolution of GPC traces during melt blending of PLA/PHB composite filled with 1 wt % of keratin at 175 °C; samples were taken after 1, 3, 6 and 10 min of blending. Inset shows evolution of *M*_w_ during the melt blending.

Degradation of the composites was followed using GPC. The GPC traces of original polymers, PLA and PHB, are shown in Figure 9a, together with spectra of prepared PLA/PHB composite containing 1 wt % of keratin. Since the big difference of molar mass of PLA (Mw = 70 kg/mol) and PHB (Mw = 270 kg/mol), in the GPC traces of the PLA/PHB composite a strong peak from PLA is accompanied with a small tail toward shorter elution volume (higher molar masses) from PHB. A shift of the PLA peak toward lower molar masses can also be seen, indicating degradation during the melt blending. In order to evaluate possible degradation of the polymers during the melt blending with keratin composites in plasticorder at 175 °C, the samples were taken and characterized by GPC at various times of blending of PLA, PHB and 1 wt % of keratin. As can be seen from the Figure 9b, a progressive decrease in molar mass was occurred during the 10 min of melt blending. The molar masses of the blend and all keratin composites after 10 min of melt blending can be compared from Table 1. A similar extent of molar mass decrease was observed for pure PLA/PHB blend and for all composites with keratin loading up to 10 wt %. Contrary to that, at the increased keratin concentration the extent of degradation was increased. At higher keratin concentration, also the concentration of functional groups of the keratin is increased, thus increasing the probability of their involvement in the degradation process.

**Figure 10:**
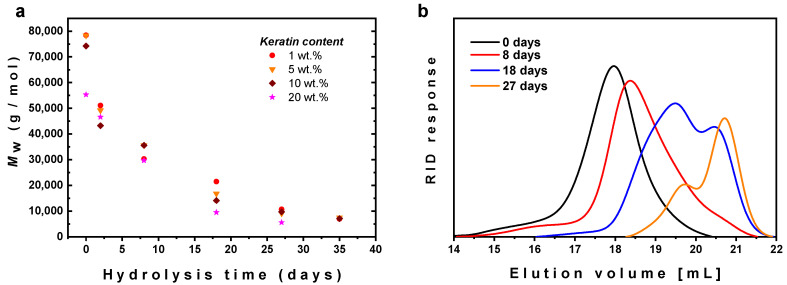
(**a**) Dependence of mass average molar mass of PLA/PHB/ATBC blends with various Keratin loading on time of hydrolysis and (**b**) evolution of GPC traces for PLA/PHB/ATBC blend with 20 wt % keratin with time of hydrolysis. The hydrolysis was performed in water vapor at 60 °C.

Finally, hydrolytic degradation of the PLA/PHB/ATBC/keratin composites at water vapor at 60 °C was investigated and followed by GPC. It should be pointed that, without considering additional effect of biodegradation, this temperature is close to temperature common in compost, therefore the rate of hydrolysis at this temperature can provide approximate information about the rate of degradation of the material under real composting conditions. As can be seen from Figure 10a, the hydrolysis was only negligibly dependent on the keratin content and after about 1 month the molar masses decreased below 10 kg/mol. These results are in good agreement with recently published hydrolysis of commercially available PLA/PHB blend unfilled or filled with 1 wt % of carbon black [17].

Evolution of the GPC traces with time of hydrolysis, shown in Figure 10b for PLA/PHB/ATBC blend containing 20 wt % of the keratin, confirmed progressive shift of both the main peak attributed to PLA and high molar mass shoulder attributed to PHB, indicating simultaneous degradation of both polymers, i.e., PLA and PHB.

## 3. Materials and Methods

Commercial poly(lactic acid) pellets PLA 4042 D were supplied from Resinex Slovakia and manufactured by Nature Works^®^ with density 1.25 g/cm^3^. Poly (3-hydroxybutyrate) PHB Biomer^®^ powder was provided by Biomer (Krailling, Germany) with density 1.20 g/cm^3^. The acetyl tributyl citrate (ATBC) 98%, was purchased from Sigma ALDRICH (Saint Louis, MO, USA). The hydrolysed keratin was prepared by procedure described previously [48] and supplied by VIPO, a.s. Partizanske, Slovakia. The PLA/PHB 85:15 blend, PLA/PHB 85:15 composites with 1 or 10 wt % of keratin and PLA/PHB/ATBC 85:15:15 composites with 1, 3, 5, 10 or 20 wt % of the keratin were prepared by melt mixing in plastic order Brabender at 175 °C, at 40 rpm (rounds per minute) for 10 min. Slabs (1 mm thick) were prepared by compression molding at 180 °C in two steps, first 2 min without pressure and additional 1 min under pressure at 2.65 MPa. Subsequently the prepared sheets were slowly cooled down under the same pressure. The specimens with rectangular shapes of “dog bone” with dimensions of 75 × 4 mm (×12.5 mm in head) for mechanical testing, strips with dimension 30 × 5 mm for dynamical mechanical thermal analysis, and discs with a diameter of 20 mm for rheology measurements were cut and stored before the measurements for approximately 24 h at ambient conditions.

The tensile tests were performed at room temperature using a Dynamometer Instron 4301 (Instron Corporation, Norwood, MA, USA) in accordance with standard ASTM D638. Seven dog-bone testing specimens for each formulation were cut for the compression molded slabs with the dimensions of the tested area of 3.5 × 30 mm with a thickness of 1 mm. A testing rate of 1 mm/min was applied until 1% deformation was reached, and then the rates were increased to 50 mm/min. Average values of the tensile strength (*σ_TS_*), elongation at break (*ε_B_*), and Young’s modulus (*E*) were determined from the stress–strain curves.

Dynamic mechanical thermal analysis (DMTA) was performed using the Dynamic Mechanical Analyser DMA Q800 (TA Instruments, New Castle, DE, USA) within the temperature range from −20 °C to 160 °C with a heating rate of 3 °C per min. The measurements were carried out in tensile mode at 1 Hz frequency with deformation amplitude of 40 µm. The storage modulus (*E*′), loss modulus (*E*″), and loss tangent (tan *δ* = *E*′/*E*″) were determined for at least three specimens of each sample formulation.

The rheological measurements of composites as well as neat PLA/PHB blend were carried out using a Rheometer AR2000 (TA Instruments, New Castle, DE, USA) at 180 °C in oscillatory mode using a 20 mm parallel plate geometry in the frequency range 0.1–100 Hz. The amplitude of deformation was 1–10% being in the linear viscoelastic region as revealed by an amplitude sweep.

The morphology of films prepared by melt mixing was observed by scanning electron microscopy (SEM) by using JSM Jeol 6610 microscope (Jeol Ltd., Tokyo, Japan) at accelerated voltage 15 kV. The samples were sputtered with a thin layer of gold. FTIR-ATR (Fourier transform infrared attenuated total reflectance spectroscopy) was applied for investigation of the surface chemical composition. The ATR-FTIR measurements were performed using a NICOLET 8700TM FTIR spectrometer (ThermoFisher Scientific, Madison, WI, USA) with a resolution of 4 cm^−1^, a scan range of 4000–400 cm^−1^.

The molar masses were estimated by gel permeation chromatography (GPC) using trifluoroethanol (TFE) as an eluent with addition of 1M potassium trifluoroacetate for ionic strength increasing. The GPC system consists of Shimadzu LC-20 pump, Shimadzu refractive index detector (Kyoto, Japan) and two PPS PFG 5 µm columns (d = 8 mm, l = 300 mm; 500 Å + 10^5^ Å) at 25 °C. Polymethyl methacrylate standards (PSS, Mainz, Germany) were used for calibration. For GPC the samples were dissolved in mixture of 1,1,1,3,3,3-hexafluoro-isopropanol (HFIP) and TFE with *v*/*v* ratio 1/9. First, 2 mg of PHB sample was treated with 100 µL of HFIP to disrupt crystalline structure of PHB followed by addition of 900 µL of TFE.

## 4. Conclusions

PLA/PHB composites filled with various keratin loading were prepared either with or without addition of ATBC as a plasticizer. It was found that addition of ATBC increased the elongation at break of about 1 to 2 orders of magnitude depending on the keratin loading. The elongation at break progressively decreased with keratin loading, while at the highest loading of 20 wt % the elongation at break was about 140%, still sufficiently high for wide range of applications. Investigation of the dynamic moduli changes using viscoelastic measurements showed higher storage moduli and lower tan delta values, indicating the more stiff but flexible behavior for the keratin composites compared to the pure PLA/PHB blend. Rheology measurements showed that addition of the keratin leads to decline in shear moduli and complex viscosity values resulting in significantly improved ductility of PLA/PHB/keratin composites. The addition of keratin up to 10 wt % did not negatively affect degradation of the PLA/PHB blend during melt blending. Hydrolytic degradation under conditions close to the ones in compost was not affected by keratin loading, and after about 1 month of hydrolysis the polymers were degraded to polymer chains with molar masses below 10,000 g/mol. The presented results clearly showed the possibility to partially replace the commonly used biodegradable polymers such as PLA and PHB by natural waste material such as acid-hydrolyzed keratin, while good mechanical properties can be achieved by using ATBC as plasticizer.

## Figures and Tables

**Table 1:** Mass weight molar masses of PLA/PHB blend and all prepared keratin composites.

	Keratin Content	Mass Weight Molar Mass (kg/mol)					
Polymer Blend		0 wt %	1 wt %	3 wt %	5 wt %	10 wt %	20 wt %
PLA/PHB		75.4	74.2	NA ^1^	NA ^1^	72.9	NA ^1^
PLA/PHB/ATBC		NA ^1^	78.4	76.7	78.3	74.2	55.3

^1^ NA stays for data not available.
